# Double-checking chromosome segregation

**DOI:** 10.1083/jcb.202301106

**Published:** 2023-04-05

**Authors:** Helder Maiato, Sónia Silva

**Affiliations:** 1 Chromosome Instability & Dynamics Group, i3S - Instituto de Investigação e Inovação em Saúde, Universidade do Porto, Porto, Portugal; 2 Instituto de Biologia Molecular e Celular, Universidade do Porto, Porto, Portugal; 3 Department of Biomedicine, Cell Division Group, Faculdade de Medicina, Universidade do Porto, Porto, Portugal

## Abstract

Enduring chromosome segregation errors represent potential threats to genomic stability due to eventual chromosome copy number alterations (aneuploidy) and formation of micronuclei—key intermediates of a rapid mutational process known as chromothripsis that is found in cancer and congenital disorders. The spindle assembly checkpoint (SAC) has been viewed as the sole surveillance mechanism that prevents chromosome segregation errors during mitosis and meiosis. However, different types of chromosome segregation errors stemming from incorrect kinetochore–microtubule attachments satisfy the SAC and are more frequent than previously anticipated. Remarkably, recent works have unveiled that most of these errors are corrected during anaphase and only rarely result in aneuploidy or formation of micronuclei. Here, we discuss recent progress in our understanding of the origin and fate of chromosome segregation errors that satisfy the SAC and shed light on the surveillance, correction, and clearance mechanisms that prevent their transmission, to preserve genomic stability.

## Teaser

Maiato and Silva discuss the origin and fate of chromosome segregation errors that satisfy the spindle assembly checkpoint, focusing on anaphase surveillance/correction mechanisms and post-mitotic clearance pathways.

## Introduction

300 billion cell divisions must take place every day in the human body to ensure tissue homeostasis and function (Sender and Milo, 2021). In vitro studies with human primary or non-transformed cells, as well as with patient-derived organoids from healthy tissue, determined that a potential chromosome missegregation event occurs once every 100–1,000 mitotic divisions (Bolhaqueiro et al., 2019; Cimini et al., 2002; Cimini et al., 2001; Crasta et al., 2012; Worrall et al., 2018), a figure that may increase more than 100-fold in chromosomally unstable cancer cells (Bakhoum et al., 2009a; Bakhoum et al., 2014; Bakhoum et al., 2009b; Bolhaqueiro et al., 2019; Crasta et al., 2012; Thompson and Compton, 2011). Even if one assumes that this represents an overestimation due to cell culture-induced artifacts (Knouse et al., 2018) and reduces the odds by few orders of magnitude (e.g. 1/120,000 cell divisions, as in *Saccharomyces cerevisiae* [Hartwell and Smith, 1985]), these numbers suggest that healthy humans live under the constant threat of gaining or losing chromosomes during somatic cell division, a condition known as aneuploidy. Indeed, low levels of aneuploidy have been detected from the very first mitotic division during human embryonic development (Currie et al., 2022) and across normal somatic tissues in humans and mammalian models (Knouse et al., 2014). Aneuploidy is also frequent in the germline (Hassold et al., 2007; Hassold and Hunt, 2001) due to a high chromosome missegregation rate during (female) meiosis in mammals (Holubcová et al., 2015; Kitajima et al., 2011).

Somatic cell aneuploidy is implicated in tumorigenesis, genomic instability, tumor evolution, metastasis, drug resistance, and reduced cancer patient survival, whereas germline aneuploidy directly accounts for infertility, pregnancy loss, and developmental disorders (Bakhoum et al., 2018; Crasta et al., 2012; Hassold and Hunt, 2001; Ippolito et al., 2021; Lee et al., 2011; Lukow et al., 2021; Replogle et al., 2020; Umbreit et al., 2020; van Dijk et al., 2021; Watkins et al., 2020; Weaver et al., 2007). However, it should be noted that the worldwide frequency of human cancers at any given moment is relatively low, currently with a total incidence of ∼1% (Global Burden of Disease Collaborative Network, 2021). Moreover, chromosome missegregation events arising during female meiosis result in less than 0.5% aneuploid liveborn (Hassold et al., 2007; Hassold and Hunt, 2001). This indicates that, despite the high frequency of potential chromosome missegregation events, aneuploidy only rarely leads to disease, supporting the existence of active surveillance, correction, and clearance mechanisms that ensure chromosomal stability during most of our lifetime and over consecutive generations, with possible evolutionary implications in the emergence of modern humans (Mora-Bermúdez et al., 2022).

**Figure 1.**
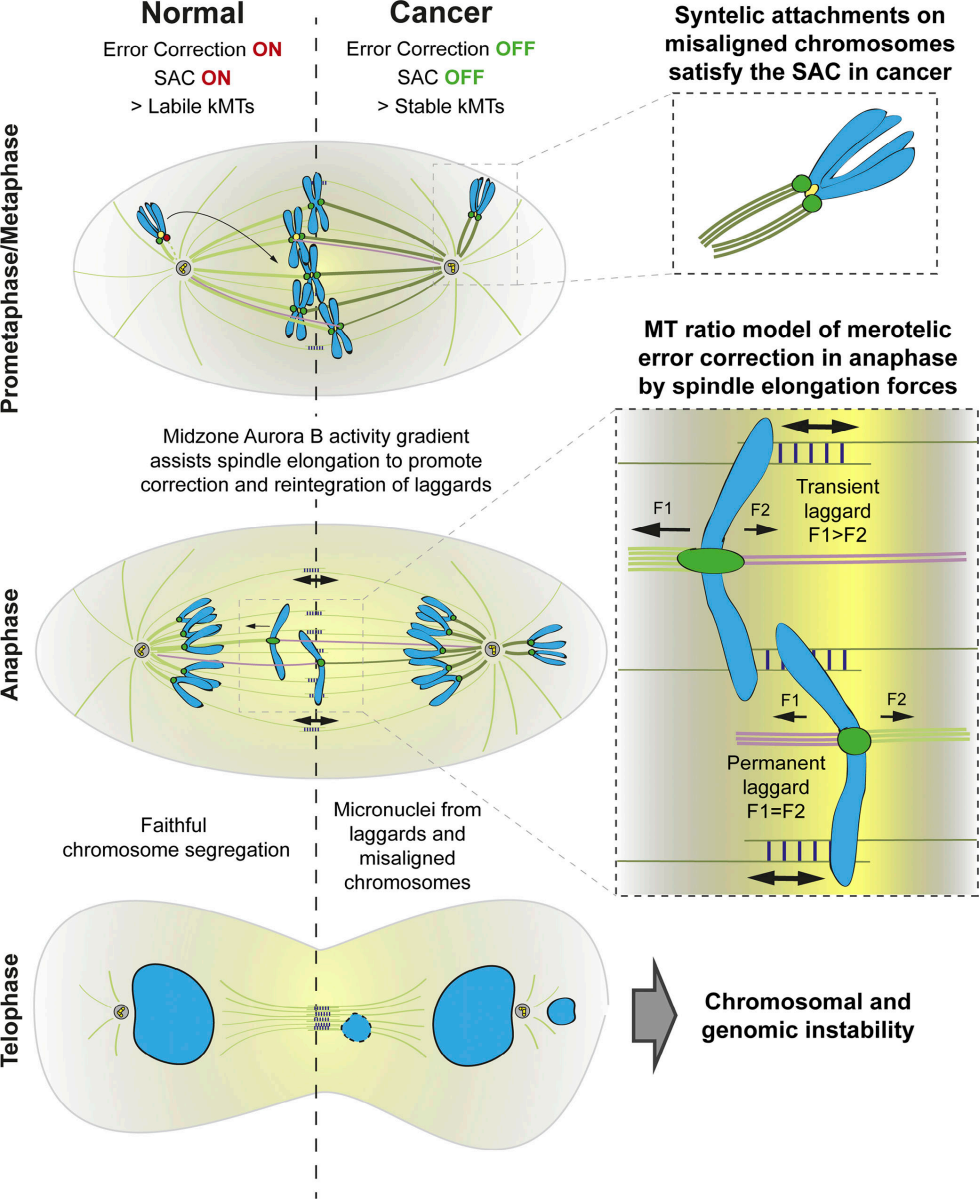
**How cells deal with chromosome segregation errors.** In normal cells, chromosome biorientation and proper kinetochore–microtubule (KT-MT) attachments are ensured by the SAC and a tension-dependent correction mechanism operated by Aurora B at centromeres throughout prometaphase and metaphase. In certain contexts, like cancer, some merotelic and syntelic KT-MT attachment errors can evade the correction machinery and satisfy the SAC, which may lead to anaphase laggards or misaligned chromosomes, respectively. Most anaphase laggards are transient and are proposed to result from unbalanced merotelic attachments. In these transient laggards, the differential transmission of forces generated by spindle elongation (F1 > F2) will stretch and elongate the kinetochore eventually, favoring segregation to the correct daughter cell, thus preventing chromosomal and genetic instability. Nevertheless, some merotelic attachments can be balanced (equal ratio of MTs toward each pole) and give rise to persistent laggards. The establishment of an Aurora B activity gradient at the spindle midzone in anaphase assists in spindle elongation, promotes the correction of attachment errors, and prevents aneuploidy and the formation of micronuclei. kMT, kinetochore microtubule.

Cell cycle checkpoints are constitutive feedback control mechanisms that delay cell cycle progression until completion of a critical event, providing time for the correction of potential errors (Hartwell and Weinert, 1989). Elimination of checkpoints relieves this dependency, allowing cells to progress through the cell cycle in the presence of potentially deleterious errors, eventually compromising cell viability or assisting cell transformation. Thus, checkpoints are often seen as non-essential pathways that only become evident in the presence of errors or perturbations that prevent checkpoint satisfaction (Khodjakov and Rieder, 2009). Most chromosome segregation errors in mitosis and meiosis are avoided by the action of the spindle assembly checkpoint (SAC) that monitors the presence of unattached kinetochores (Lara-Gonzalez et al., 2021; Rieder et al., 1995; Rieder et al., 1994; Touati and Wassmann, 2016). In addition, a tension-dependent correction mechanism involving Aurora B kinase at centromeres promotes chromosome biorientation prior to anaphase (Lampson and Grishchuk, 2017; Lampson et al., 2004; Nicklas and Ward, 1994; Fig. 1). However, because SAC satisfaction occurs within the framework of individual kinetochores and is independent of opposing pulling forces (O’Connell et al., 2008; Uchida et al., 2021), some kinetochore–microtubule attachment errors may evade the correction machinery. This is the case of merotelic attachments (when a single kinetochore attaches to microtubules oriented to both spindle poles) that, if uncorrected, may lead to anaphase lagging chromosomes (laggards), or syntelic attachments (in which both kinetochores of a misaligned chromosome are oriented toward the same spindle pole; Fig. 1). How cells deal with enduring chromosome attachment errors that satisfy the SAC and might lead to missegregation remains an exciting fundamental question with broad clinical implications.

## Mechanism of anaphase error correction

Recent high-resolution live-cell studies tracking kinetochore or chromosome behavior through metaphase and anaphase in human somatic cells in culture have revealed that many more chromosomes tend to lag behind in anaphase than previously anticipated (Orr et al., 2021; Sen et al., 2021). This behavior is, at least in part, due to prevailing merotelic attachments and can be predicted from their oscillatory pattern during metaphase (Cimini et al., 2004; Sen et al., 2021). Importantly, most laggards have a transient nature and resume poleward motion, suggesting an active error correction mechanism that prevents missegregation. The existence of an anaphase error correction mechanism was originally deduced from the relatively high number of merotelic attachments in metaphase that persist through anaphase and the much lower number of enduring laggards (Cimini et al., 2004; Cimini et al., 2003). This led to a model based on the microtubule ratio at kinetochores that explains how spindle elongation during anaphase prevents potential segregation errors derived from merotelic attachments (Cimini et al., 2004; Fig. 1).

The role of anaphase spindle mechanics was first established through the observation that merotelic kinetochores with uneven microtubules facing the poles experience a significant stretch and elongate under tension, eventually favoring segregation to the correct daughter cell. Only a small fraction (<10%) of the merotelic kinetochores that persist through anaphase show an even microtubule ratio facing the poles and result in long-lasting lagging chromosomes that missegregate (Cimini et al., 2004). Live imaging of mouse oocytes undergoing mitosis-like meiosis II also revealed that >20% of kinetochore–microtubule attachments are merotelic or lateral at metaphase II, whereas only <1% of all chromosomes lag behind during anaphase II (Kouznetsova et al., 2019). Most striking, less than 10% of all lagging chromosomes missegregate and give rise to aneuploid gametes. This is also the case in insect spermatocytes where most lagging chromosomes resulting from unbalanced merotelic attachments correct during anaphase II and reintegrate in the main nuclei (Janicke et al., 2007).

More recently, it was demonstrated that spindle elongation is directly required for anaphase error correction (Orr et al., 2021). The acute inactivation of the antiparallel microtubule sliding motor kinesin-5 after SAC satisfaction and anaphase onset showed that the attenuated anaphase spindle elongation increased the frequency of anaphase cells with lagging chromosomes and a twofold increase in the number of lagging chromosomes per cell relative to controls. Thus, anaphase error correction appears to prevent aneuploidy from enduring kinetochore–microtubule attachment errors that satisfy the SAC during mitosis and meiosis in metazoans, but the underlying mechanism remains a matter of debate.

Aurora B kinase activity at centromeres plays a key role in error correction during early mitosis (Lampson and Grishchuk, 2017). However, within ∼1 min after anaphase onset, Aurora B leaves the centromere and is actively transported by the kinesin-6 Mklp2 toward the spindle midzone (Adriaans et al., 2020; Gruneberg et al., 2004; Murata-Hori et al., 2002; Orr et al., 2021), where it establishes a phosphorylation gradient on chromosome, kinetochore, and spindle microtubule substrates (Fuller et al., 2008; Papini et al., 2021; Sen et al., 2021; Tan and Kapoor, 2011). Inhibition of Aurora B activity or prevention of its relocation to the spindle midzone during metaphase (Sen et al., 2021), as well as its acute inhibition at anaphase onset (Orr et al., 2021), resulted in a significant increase in laggards. While it could be argued that Aurora B inhibition during metaphase might compromise ongoing correction of merotelic attachments prior to anaphase onset (Cimini et al., 2003; Cimini et al., 2006; Knowlton et al., 2006), its acute inhibition or prevention of its relocation at anaphase onset strongly suggests that Aurora B activity at the spindle midzone is either required to avoid new attachment errors or for anaphase error correction. As all kinetochores must be attached to microtubules to satisfy the SAC, and because attached microtubules experience little or no turnover during anaphase (Gorbsky and Borisy, 1989; Zhai et al., 1995), it is unlikely that new attachment errors take place during anaphase, favoring the anaphase error correction hypothesis.

**Figure 2.**
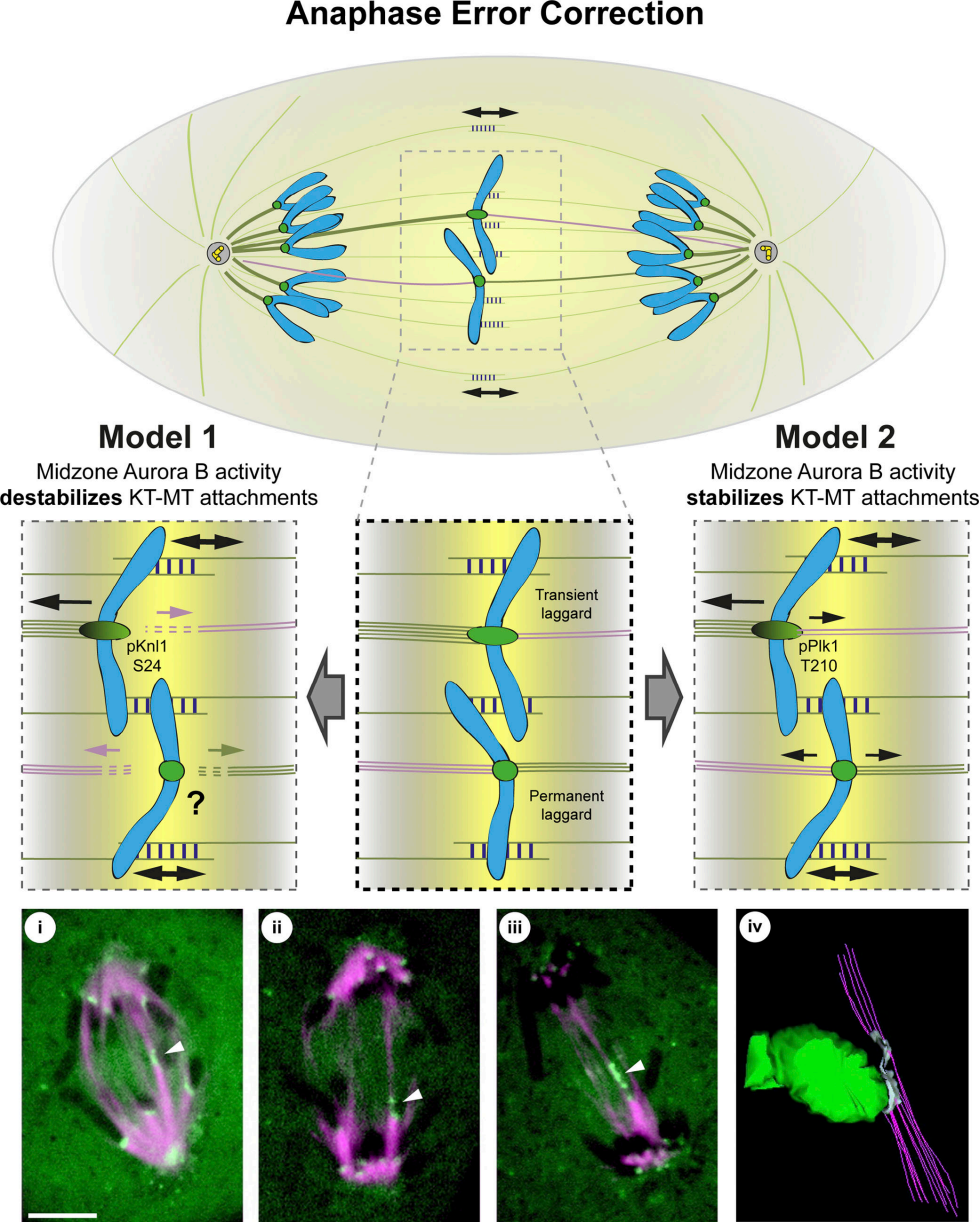
**Models for anaphase error correction mediated by an Aurora B activity gradient at the spindle midzone.** Model 1: Upon leaving the centromeres, Aurora B establishes an activity gradient at the midzone and phosphorylates specific kinetochore substrates, like pKnl1 (Ser24), leading to the destabilization of kinetochore–microtubule (KT-MT) attachments. In the presence of unbalanced merotelic attachments, microtubules on the incorrect side would efficiently be destabilized. This model cannot explain the persistence of KT-MT attachments seen experimentally in permanent laggards at the spindle midzone (balanced merotelic attachments). Model 2: Midzone Aurora B activity promotes anaphase error correction of merotelic kinetochores by stabilizing KT-MT attachments, evidenced by the accumulation of marks such as pPlk1 (Thr210). This stabilization would assist in the mechanical transmission of spindle elongation forces to promote the correction of unbalanced merotelic attachments on transient lagging chromosomes. Balanced merotelic attachments would be expected to result in permanent laggards that remain attached with microtubules, in line with observations in living PtK1 cells and 3D electron microscopy reconstructions (i–iv; modified with permission from Salmon et al., 2005). Microtubules (magenta) and kinetochores (green) are depicted. White arrowheads indicate permanent or transient merotelic attachments. Scale bar is 5 μm.

Two possible models have been proposed to explain how a midzone-based Aurora B activity gradient mediates error correction during anaphase (Fig. 2). One is based on the well-established microtubule destabilizing roles of Aurora B at centromeres during early mitosis (Lampson and Grishchuk, 2017) and proposes that, upon leaving the centromeres, a midzone Aurora B activity gradient promotes the phosphorylation of kinetochore substrates to destabilize microtubule attachments (Sen et al., 2021). In support of this model, Aurora B–mediated phosphorylation of Knl1 on Ser24, which was shown to destabilize kinetochore–microtubule attachments and prevent the kinetochore recruitment of PP1 phosphatase early in mitosis (Liu et al., 2010; Welburn et al., 2010), was enriched on the midzone-facing side of kinetochores from lagging chromosomes (Sen et al., 2021). Aurora B at the spindle midzone would promote faithful segregation by destabilizing microtubules on the incorrect side more efficiently. In agreement, upon Aurora B inhibition, the majority of anaphase kinetochores remained stretched for an extended period. Assuming that midzone Aurora B activity indeed destabilizes kinetochore–microtubule attachments, this model explains how “unbalanced” merotelic attachments (i.e., with uneven microtubule attachments facing each pole) may be corrected during anaphase. Nevertheless, this model falls short in explaining why “balanced” merotelic attachments that remain right at the peak of Aurora B activity at the spindle midzone do not detach and missegregate (Cimini et al., 2004).

An alternative model favors the idea that midzone Aurora B activity mediates anaphase error correction of merotelic kinetochores by assisting the mechanical transmission of spindle forces to a stable kinetochore–microtubule interface on lagging chromosomes (Orr et al., 2021). Live-cell imaging studies of kinetochore microtubules in epithelial rat kangaroo PtK1 cells and insect spermatocytes undergoing meiosis II have shown that neither “balanced” nor “unbalanced” merotelic attachments ever detach during correction in anaphase (Cimini et al., 2004; Janicke et al., 2007), suggesting that the mechanism of anaphase error correction does not require microtubule detachment from kinetochores. In agreement, error correction by detachment of kinetochore microtubules requires high Cdk1 activity (Vázquez-Novelle et al., 2014), which decreases sharply during metaphase and throughout anaphase (Afonso et al., 2019; Clute and Pines, 1999). Moreover, kinetochores experiencing sustained tension were recently shown to suppress Aurora B–mediated microtubule release and never detach, with the respective kinetochore-fibers (k-fibers) undergoing persistent microtubule polymerization (de Regt et al., 2022; Long et al., 2020). Importantly, Aurora B function at centromeres and spindle midzone can be uncoupled (Lens et al., 2006), and previous works have implicated Aurora B in the stabilization of midzone microtubules during anaphase to support efficient spindle elongation (Ferreira et al., 2013; Murata-Hori et al., 2002; Nunes Bastos et al., 2013; Uehara et al., 2013). Thus, Aurora B might assist anaphase error correction both by stabilizing kinetochore–microtubule attachments and by regulating spindle elongation.

Direct measurements of kinetochore–microtubule half-life during anaphase revealed a low kinetochore–microtubule turnover (Gorbsky and Borisy, 1989; Zhai et al., 1995), which was found to depend on Aurora B localization at the spindle midzone and, consequently, its absence from centromeres (Orr et al., 2021). However, these measurements were not directly performed on lagging chromosomes with merotelic attachments. Despite this caveat, partial destabilization of kinetochore–microtubule attachments significantly increased the frequency of anaphase cells with lagging chromosomes that were unable to be corrected (Orr et al., 2021). Moreover, phosphorylation/activation of Plk1 at Thr210, an indicator of stable kinetochore–microtubule attachments during early mitosis (Liu et al., 2012) that is thought to be regulated by Aurora B (Carmena et al., 2012), was specifically enriched at kinetochores of anaphase lagging chromosomes induced by the formation of merotelic attachments (Orr et al., 2021). The microtubule-associated protein Astrin that decorates stable end-on kinetochore–microtubule attachments (Mack and Compton, 2001; Manning et al., 2010) was also found enriched at anaphase kinetochores proximal to the spindle midzone, including on lagging chromosomes, in an Aurora B–dependent manner (Papini et al., 2021). Phosphorylation of two additional Aurora B substrates at kinetochores, Dsn1 on Ser109 and CENP-A on Ser7, also follows a gradient pattern centered on Aurora B at the spindle midzone during anaphase (Papini et al., 2021). In particular, Dsn1 phosphorylation on Ser109 was proposed to regulate kinetochore disassembly as cells progress through anaphase (Papini et al., 2021). Altogether, these data are at odds with models envisioning a destabilizing role by Aurora B at the spindle midzone and provide evidence that anaphase error correction requires stable kinetochore–microtubule attachments. As even subtle phosphorylation changes on key molecules that regulate kinetochore–microtubule attachment stability depend on Aurora B and Cdk1 activity (Kucharski et al., 2022), future investigation should focus on understanding how the cumulative phosphorylation of different Aurora B substrates with potentially opposing roles at kinetochores impacts overall kinetochore function during anaphase (when Cdk1 activity is low). This knowledge will be necessary to understand how a midzone Aurora B activity gradient mediates anaphase error correction and to test whether similar mechanisms operate during meiosis. Moreover, because Aurora B is overexpressed in highly aneuploid cancers (Pfister et al., 2018; Smith et al., 2005; Takeshita et al., 2013) and its overexpression was recently shown to inhibit Aurora B activity toward different kinetochore substrates (Britigan et al., 2022), it will be important to investigate whether anaphase surveillance and error correction mechanisms are disrupted in human cancers.

## Coordination of anaphase error correction with nuclear envelope reassembly: Passive unsupervised control or active surveillance?

**Figure 3.**
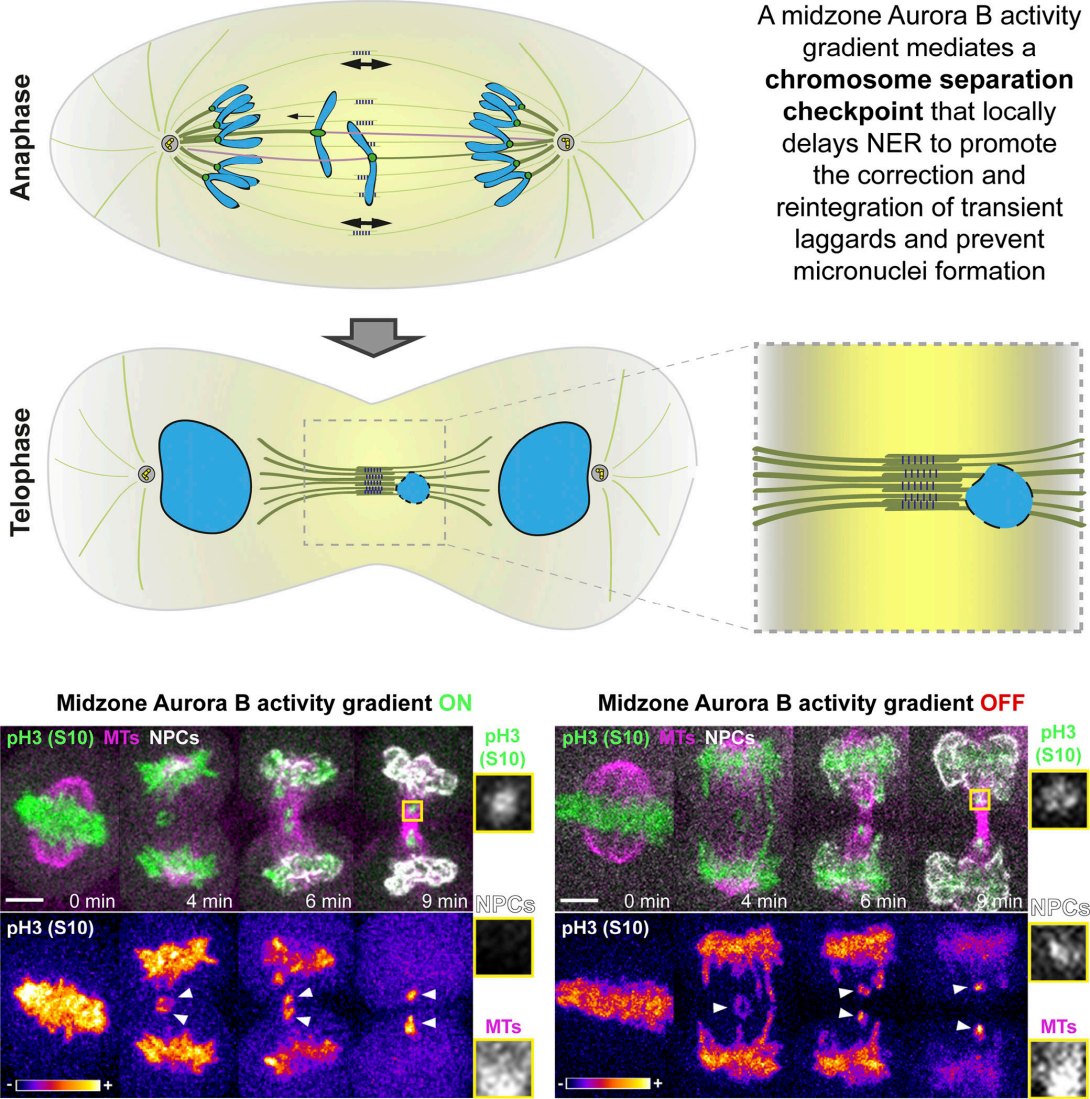
**Role of Aurora B in anaphase error correction and the spatiotemporal coordination of mitotic exit.** The midzone-based Aurora B activity gradient was proposed to establish a chromosome separation checkpoint that delays chromosome decondensation and NER on incompletely separated chromosomes, including laggards. This allows their correction and reintegration into the main nuclei (Aurora B activity gradient ON). By inhibiting Aurora B activity or preventing its midzone localization, NER is initiated synchronously on all chromosomes, regardless of separation (Aurora B activity gradient OFF), independently of the presence of midzone/midbody microtubule bundles. White arrowheads indicate lagging chromosomes. Time-lapse series was modified from Orr et al. (2021). Scale bar is 5 μm. MT, microtubule.

The finding of a distinct phosphorylation state on Aurora B substrates at kinetochores of anaphase lagging chromosomes (Fuller et al., 2008; Orr et al., 2021; Papini et al., 2021; Sen et al., 2021) suggests that the mechanism underlying anaphase error correction involves active signaling. Phosphorylation of Aurora B substrates on chromosomes/chromatin, such as Histone H3 on Ser10, has long been known to decrease as a function of the distance migrated by chromosomes during anaphase (Afonso et al., 2014; Fuller et al., 2008; Orr et al., 2021). While the functional significance of Histone H3 phosphorylation on Ser10 remains unclear, a midzone-based Aurora B activity gradient on chromosomes was proposed to mediate a chromosome separation checkpoint that delays chromosome decondensation and nuclear envelope reassembly (NER) in response to incomplete chromosome separation during anaphase in metazoans, including humans (Afonso et al., 2014; Maiato et al., 2015; Fig. 3). This spatiotemporal control of NER ensures that the nuclear envelope does not form prematurely on separating chromosome masses during normal anaphase, contributing to the correct segregation into two euploid daughter cells. In addition, spatiotemporal control of NER is particularly evident on anaphase lagging chromosomes, which show a delay in NER relative to normally separating chromosomes in the same cell (Afonso et al., 2014; de Castro et al., 2017; Liu et al., 2018; Orr et al., 2021). Importantly, even on normally separating chromosome masses, NER is asymmetric, starting on the pole-facing side as the chromosomes approach the poles while remaining “open” toward the midzone until late anaphase (Güttinger et al., 2009). Most striking, the dependence of NER on chromosome separation during anaphase can be experimentally relieved by inhibiting Aurora B activity at anaphase onset (Afonso et al., 2014; Liu et al., 2018) or by preventing its association with the spindle midzone, causing all chromosomes, including laggards, to simultaneously initiate NER (Afonso et al., 2014; Orr et al., 2021).

But why is the spatiotemporal control of NER important? On one hand, preventing premature NER on incompletely separated chromosomes during normal anaphase protects against polyploidy. Whole-genome duplication is a common evolutionary event that is also frequent in >30% of human tumors early in tumorigenesis and is thought to promote chromosomal instability and metastasis by providing a selective advantage in certain contexts (Ben-David and Amon, 2020; Bielski et al., 2018; Dewhurst et al., 2014; Gemble et al., 2022; Newcomb et al., 2021; Prasad et al., 2022; Priestley et al., 2019; Storchova and Pellman, 2004; Zack et al., 2013). On the other hand, spatiotemporal control of NER would allow the correction of anaphase lagging chromosomes and prevent formation of micronuclei (Afonso et al., 2014). Micronuclei are well-established genotoxicity biomarkers that have regained attention due to their causal link with chromothripsis (Fenech et al., 2020). Chromothripsis is a widespread mutational phenomenon characterized by massive genomic rearrangements, which was recently implicated in tumor evolution, acquired drug resistance, and oncogene activation, as well as a possible cause of congenital disorders (Crasta et al., 2012; Kloosterman and Cuppen, 2013; Ly and Cleveland, 2017; Shoshani et al., 2021; Stephens et al., 2011; Zhang et al., 2015). Thus, spatiotemporal control of NER prevents polyploidy, aneuploidy, and possible downstream genomic rearrangements, allowing chromosomes to separate and error correction to take place during anaphase. But whether or not this involves active surveillance or is passively unsupervised remains controversial.

One may argue that a delay of just a few minutes in a process that is normally completed within 5–10 min in human somatic cells cannot be explained by a robust checkpoint mediated by Aurora B at the spindle midzone. Instead, midzone microtubules may act independently of Aurora B activity by forming a physical barrier that selectively prevents the recruitment of non-core nuclear envelope proteins (those targeted to the chromosome peripheral regions during NER [Kutay et al., 2021]), including nuclear pore complex (NPC) proteins, (Liu et al., 2018; Fig. 3). Thus, completion of NER on lagging chromosomes would be strictly dependent on the disassembly of midzone microtubules as cells exit mitosis, with irreversible nuclear envelope defects on lagging chromosomes emerging as an unsupervised pathological condition that inevitably links mitotic errors to chromothripsis (Liu et al., 2018; Liu and Pellman, 2020). However, the formation of a functional spindle midzone depends on Aurora B activity (Ferreira et al., 2013; Murata-Hori et al., 2002; Nunes Bastos et al., 2013; Uehara et al., 2013). Moreover, dynamic microtubules are necessary for Aurora B accumulation at the spindle midzone and to increase Aurora B activity toward several microtubule-associated substrates (Murata-Hori et al., 2002; Noujaim et al., 2014; Wheatley et al., 2001), making it difficult to distinguish a “passive” role of microtubules, from “active” microtubule-dependent Aurora B–mediated signaling.

In an attempt to separate the role of microtubules from that of Aurora B at the spindle midzone in the spatiotemporal control of NER, high-resolution live-cell microscopy was used to simultaneously monitor Aurora B activity on chromosomes, the recruitment of NPC proteins, and the distribution of spindle midzone microtubules throughout anaphase, upon manipulation of Aurora B midzone localization (Orr et al., 2021). These experiments suggested that a midzone-based Aurora B phosphorylation gradient, rather than midzone microtubules per se, delays the completion of NER on anaphase lagging chromosomes in human cells. Nevertheless, a minority of enduring lagging chromosomes that fail to correct during anaphase might still result in micronuclei with defective nuclear envelopes (Liu et al., 2018). Another study showed that micronuclei that arise from mitotic slippage in the absence of microtubules fail to properly localize lamin B1 to the nuclear envelope, thereby promoting micronuclear rupture and concomitant DNA damage (Kneissig et al., 2019). Thus, microtubules are neither sufficient nor required to cause nuclear envelope defects on micronuclei.

Quantitative microscopy analyses further revealed that even just a small but significant delay in the completion of NER relative to the main segregating chromosome masses allows most anaphase lagging chromosomes in human cells to gradually correct and move away from the spindle midzone. Moreover, this delay depended on the establishment of a midzone-based Aurora B phosphorylation gradient that prevented formation of micronuclei (Orr et al., 2021; Sen et al., 2021). Therefore, consistent with the transient nature of most anaphase lagging chromosomes, NER delay does not inevitably result in pathological conditions associated with micronucleus formation. This appears to be the case even upon induction of massive chromosome segregation errors by experimental abrogation of the SAC and/or by preventing the formation of a tight metaphase plate, with the vast majority of anaphase lagging chromosomes not resulting in micronuclei (Cohen-Sharir et al., 2021; Fonseca et al., 2019; Klaasen et al., 2022; Worrall et al., 2018).

Concerning potential targets involved in the spatiotemporal regulation of NER, Aurora B may regulate Condensin I removal and/or recruitment of HP1 and LBR as chromosomes separate during anaphase (Afonso et al., 2014; Giet and Glover, 2001; Lipp et al., 2007; Nakazawa et al., 2011; Ono et al., 2004; Orr et al., 2021; Tada et al., 2011; Takemoto et al., 2007; Warecki and Sullivan, 2018) or may more directly regulate the phosphorylation of lamins and NPC proteins (reviewed by Afonso et al., 2017). In parallel, Aurora B at the spindle midzone might regulate the phosphorylation of targets involved in NER by controlling residual but highly localized Cdk1 activity and counteracting PP1/PP2A phosphatase activities in space and time during anaphase (Afonso et al., 2019; de Castro et al., 2017; Holder et al., 2020; Mathieu et al., 2013; Vagnarelli, 2021; Zhou and Homer, 2022). Future work will be necessary to dissect the mechanistic underpins involved in the spatiotemporal regulation of NER by Aurora B.

## When anaphase surveillance and correction mechanisms fail—dealing with aneuploidy and micronuclei

Anaphase lagging chromosomes that escape surveillance and correction mechanisms operating in anaphase rarely missegregate to give rise to aneuploidy, but they may lead to the formation of micronuclei (Cimini et al., 2004; Thompson and Compton, 2011). Importantly, aneuploidy and micronuclei in non-transformed cells are normally poorly tolerated and have been shown to cause a p53-dependent reduction in cell proliferation/viability (Fonseca et al., 2019; Li et al., 2010; Narkar et al., 2021; Pfau et al., 2016; Sablina et al., 1998; Santaguida et al., 2017; Thompson and Compton, 2008; Thompson and Compton, 2010). This appears to involve differential phosphorylation of histone H3.3 at Ser31, an Aurora B target (Li et al., 2017), which is necessary for p53 accumulation in the nucleus of aneuploid daughter cells (Hinchcliffe et al., 2016). However, p53 activation and cell cycle arrest in response to aneuploidy do not seem to be universal and might depend on the cell type, the nature of the segregation errors, and cell culture conditions (Narkar et al., 2021; Santaguida et al., 2017; Soto et al., 2017). In the context of certain cancers where tumor suppressor genes (including p53) are frequently mutated, aneuploidy and micronuclei have been shown to increase cell fitness and proliferative potential, potentiating tumor evolution (Ben-David et al., 2014; Ly et al., 2011; Rutledge et al., 2016).

Micronuclei derived from chromosome segregation errors have four possible outcomes upon mitotic exit: (1) persistence as independent structures; (2) reincorporation into the main nucleus; (3) degradation; or (4) extrusion (reviewed in Hintzsche et al., 2017). Approximately 70% of micronuclei appear to persist as independent structures, whereas the remaining fraction disappears either by reincorporation into the main nucleus in the subsequent mitosis (Crasta et al., 2012; Hatch et al., 2013; Huang et al., 2012; Soto et al., 2018), lysosome-mediated autophagy (Bartsch et al., 2017; Rello-Varona et al., 2012; Zhao et al., 2021), or extrusion through the cytoplasmic membrane (Schriever-Schwemmer et al., 1997; Shimizu et al., 2000). For those micronuclei that persist, nuclear envelope rupture and collapse were reported to occur in up to 60% of the cases (Hatch et al., 2013). The underlying causes appear to be linked with a lower density of NPCs, defective content of lamin B1, and other essential nuclear envelope components (Crasta et al., 2012; Géraud et al., 1989; Hatch et al., 2013; Hatch and Hetzer, 2016), possibly due to delayed recruitment of non-core nuclear envelope and NPC proteins to anaphase lagging chromosomes (Afonso et al., 2014; Liu et al., 2018; Orr et al., 2021). This may expose DNA to damage during cytokinetic furrow ingression and directly lead to structural chromosome aberrations (Janssen et al., 2011). In addition, nuclear envelope defects lead to loss of compartmentalization and severely impair transport in and out of micronuclei, thus affecting the ability to undertake proper DNA repair, transcription, and replication (Crasta et al., 2012; Hatch et al., 2013; Okamoto et al., 2012).

**Figure 4.**
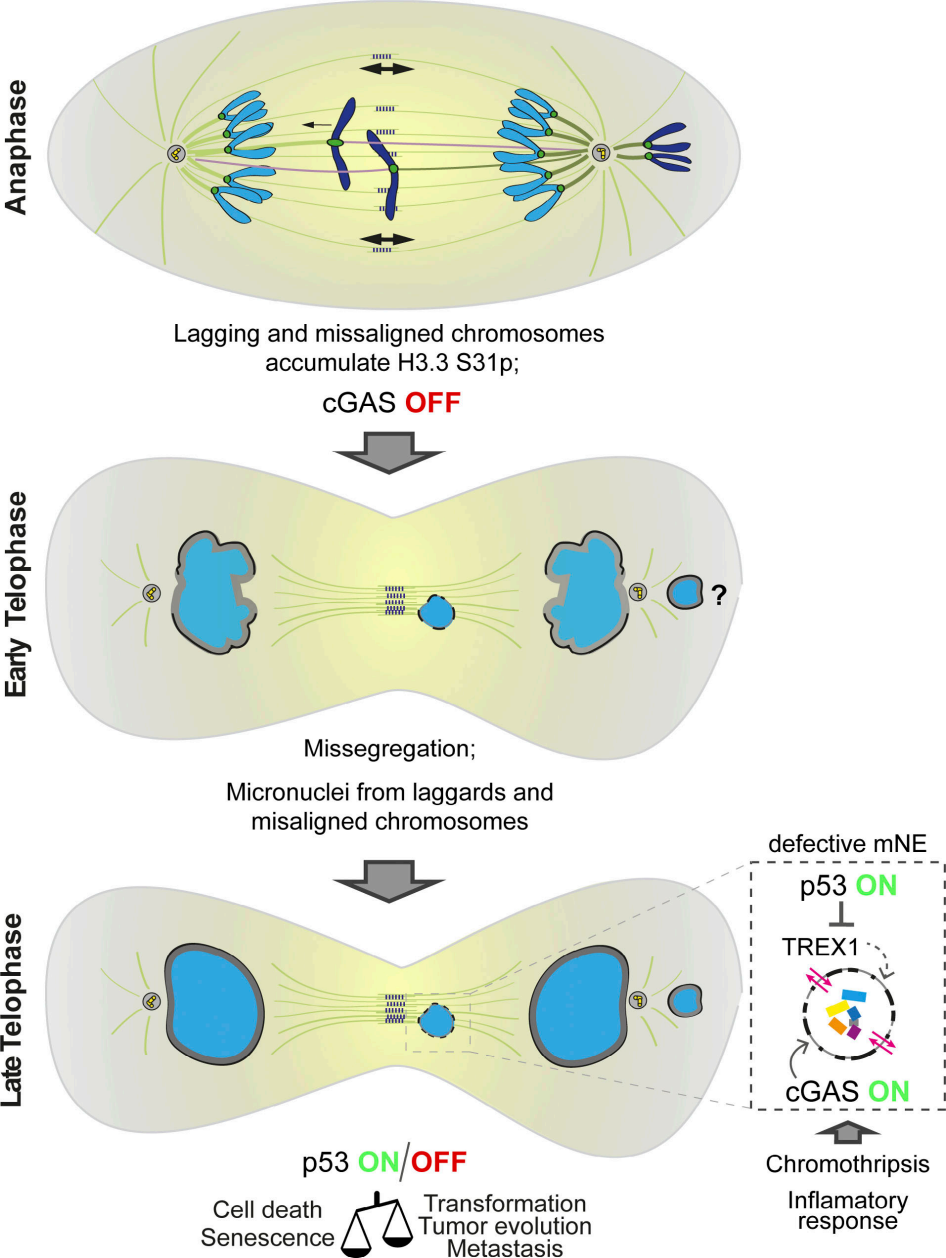
**Cellular response to aneuploidy and formation of micronuclei.** Histone H3.3, an Aurora B target, is differentially phosphorylated at Ser31 on laggards (center) and misaligned chromosomes (right) in metaphase and anaphase cells, which is necessary for p53 accumulation in the nucleus of aneuploid daughter cells. Chromosomes that do not segregate with the main chromosome mass are enclosed by nuclear envelope (NE) components in a differential manner, which can lead to defective deposition of NPCs and other non-core components to the newly formed NE. The integrity of NE on micronuclei derived from misaligned chromosomes is currently unknown. Micronuclei (mNE) with a defective NE can rupture and expose their DNA to the cytosolic environment, leading to extensive DNA damage. p53 promotes the degradation of TREX1 endonuclease via the proteasome, facilitating the activation of cGAS-STING and consequent innate immune response due to the accumulation of cytosolic dsDNA from ruptured micronuclei, which are potential hubs for chromothripsis. Aneuploidy and micronuclei in non-transformed cells cause a p53-dependent reduction in cell proliferation and viability. Alterations in the p53 status may alternatively increase fitness and proliferative potential in certain contexts, potentiating tumor evolution and metastasis.

The loss of compartmentalization on micronuclei exposes DNA to the cytosolic environment that triggers the action of cGAS, a strong double-stranded DNA (dsDNA) sensor that mounts a type-I IFN innate immune response through its adaptor protein STING, referred to as the cGAS-STING pathway (Bartsch et al., 2017; Harding et al., 2017; MacKenzie et al., 2017). The activation of the cGAS-STING pathway has been associated with mitotic cell death, senescence, and apoptosis of micronucleated cells (Harding et al., 2017; Santaguida et al., 2017; Yang et al., 2017; Zierhut et al., 2019). Interestingly, Aurora B–mediated phosphorylation of chromatin-associated cGAS was recently shown to prevent its premature activation during mitosis (Li et al., 2021). Moreover, chromatin bridges were also recently proposed to activate the cGAS-STING pathway in an Aurora B–dependent manner (Flynn et al., 2021). These findings link Aurora B–dependent surveillance and correction mechanisms operating during anaphase with those involved in the post-mitotic clearance of aneuploid and micronucleated cells to preserve genomic stability (Fig. 4).

Given the possible role of p53 in limiting the proliferation of aneuploid and micronucleated cells, an outstanding question is whether the cGAS-STING pathway somehow crosstalks with p53. Recently, a mutant form of p53 was shown to suppress innate immune signaling through the cGAS-STING pathway, resulting in immune evasion and tumor progression (Ghosh et al., 2021). In this context, p53 promoted the degradation of the DNA endonuclease TREX1 via the proteasome, resulting in the accumulation of cytosolic dsDNA from ruptured micronuclei and consequently triggering a cGAS-STING–mediated innate immune response (Ghosh et al., 2023). Thus, the tumor suppressor role of p53 might involve active signaling through the cGAS-STING pathway (Fig. 4).

While a lot remains to be known regarding the mechanisms underlying cGAS-STING–mediated immunosurveillance of mitotic errors and their respective implications for tumor evolution, defective micronuclei are now well established as potential hubs for chromothripsis (Crasta et al., 2012; Hastings et al., 2009; Kneissig et al., 2019; Liu et al., 2011; Stephens et al., 2011; Zhang et al., 2015). Chromothripsis (and other similar catastrophic events that together define chromoanagenesis) has entered the spotlight since it provides an explanation for many chromosomal abnormalities observed in human cancers (reviewed in Holland and Cleveland, 2012). Despite the initially reported low incidence in cancer cells (Cai et al., 2014; Stephens et al., 2011), technical advances in genome sequencing have revealed an astonishing frequency of nearly 30% of chromothripsis in 2,543 analyzed samples, covering 37 different cancer types (Cortés-Ciriano et al., 2020). However, it remains unclear whether micronuclei and associated chromothripsis are drivers of cancer and its evolution or a downstream consequence of yet other driving events. The fact that chromothripsis has been observed in both primary tumors and metastasis (Kloosterman et al., 2011), together with the huge variation in the frequency observed between different tumor types, with some being particularly prone and others totally refractory (Cortés-Ciriano et al., 2020; Malhotra et al., 2013), suggests that chromothripsis depends on various genetic and/or environmental factors. For instance, p53 has been associated with restricting the occurrence of chromothripsis events in some cancers (Rausch et al., 2012). Interestingly, micronuclei and chromothripsis are not necessarily malignant events and can be stably transmitted over several generations in healthy individuals (Bertelsen et al., 2016; Chiang et al., 2012; de Pagter et al., 2015; Hopf et al., 2020; Peace et al., 1999). In agreement, micronuclei resulting from loss of interchromosome compaction during anaphase in mice knockout for the kinesin-8 Kif18a, a microtubule plus-end-directed motor that suppresses microtubule polymerization (Du et al., 2010), form apparently stable nuclear envelopes and do not promote tumorigenesis (Fonseca et al., 2019; Sepaniac et al., 2021). In fact, loss of Kif18a appears to protect against tumor formation upon chemical-induced carcinogenesis (Zhu et al., 2013). Altogether, micronuclei and extreme chromosome rearrangements that may derive from them, including breakage of multiple protein-coding genes, can be tolerated and do not necessarily result in cancer. Nevertheless, chromothripsis in healthy individuals does appear to affect reproduction and increases the risk of miscarriages and severe congenital disorders, probably through problems in meiosis (Bertelsen et al., 2016; de Pagter et al., 2015).

## Final remarks and outlook

While the high rates of aneuploidy during female meiosis have been linked to a weakened SAC and intrinsically unstable kinetochore–microtubule attachments (Kitajima et al., 2011; Kyogoku and Kitajima, 2017; Touati and Wassmann, 2016; Yoshida et al., 2015), aneuploid cancer cells are known to have a robust SAC (Rieder and Maiato, 2004; Tighe et al., 2001). Thus, aneuploidy and all downstream consequences in cancer might instead be due to errors arising from incorrect kinetochore–microtubule attachments that satisfy a perfectly functioning SAC. Recent evidence also indicated that many more merotelic kinetochore–microtubule attachments than previously thought result in anaphase lagging chromosomes that satisfy the SAC in human cultured cells (Orr et al., 2021; Sen et al., 2021), but only rarely result in micronuclei, hinting at the existence of active surveillance and correction mechanisms during anaphase that we are just starting to understand. Interestingly, although most micronuclei in cancer cells derive from anaphase lagging chromosomes that rarely missegregate (Thompson and Compton, 2011), a recent study has shown that misaligned chromosomes that satisfy the SAC often directly missegregate without lagging behind in anaphase and have the highest probability to form micronuclei (Gomes et al., 2022), representing a major source of chromosomal instability in primary and metastatic breast tumors (Tucker et al., 2023; Fig. 1). In line with these findings, recent experiments in which CENP-E activity was inhibited in human cells suggest that endomembrane “ensheathing” of misaligned chromosomes might facilitate the formation of micronuclei and delay SAC satisfaction (Ferrandiz et al., 2022). Correction of erroneous attachments underlying some chromosome alignment defects (e.g., syntelic attachments) also appears to be less robust in cancer cells with overly stabilized kinetochore–microtubule attachments (Bakhoum et al., 2009a; Salimian et al., 2011; Fig. 1). Moreover, hyperstabilization of kinetochore–microtubule attachments in otherwise normal non-transformed human cells leads to SAC satisfaction in the presence of misaligned chromosomes, especially under conditions that promote the formation of syntelic attachments (Brito et al., 2008; Klaasen et al., 2022; Yang et al., 2009). Lastly, misaligned chromosomes that satisfy the SAC may also arise due to k-fiber minus-end detachment from mitotic spindle poles after biorientation and directly lead to chromosome missegregation and micronuclei (van Toorn et al., 2023). Thus, similar to chromosome non-disjunction during female meiosis, accounting for most aneuploidies in humans (Hassold et al., 2007), misaligned chromosomes that establish syntelic attachments and satisfy the SAC may represent a previously overlooked mechanism driving chromosomal/genomic instability during cancer cell division, while compromising embryonic viability.

In addition to direct missegregation from misaligned chromosomes, late-aligning chromosomes associated with peripheral nuclear positioning during interphase are also more prone to lag behind in anaphase and missegregate at higher frequencies (Klaasen et al., 2022; Kuniyasu et al., 2018). Likewise, failure to cluster parental pronuclei genomes upon fertilization has also been shown to promote chromosome congression defects (and anaphase lagging chromosomes) that give rise to micronuclei and impair embryonic development (Cavazza et al., 2021).

Overall, these recent findings instigate future studies to determine the underlying mechanisms that normally prevent aneuploidy during mitosis and meiosis, while continuing to evaluate the contribution of aneuploidy-inducing events of different origin and how cells deal with them in health and disease contexts.
